# Glucose delays age-dependent proteotoxicity

**DOI:** 10.1111/j.1474-9726.2012.00855.x

**Published:** 2012-10

**Authors:** Arnaud Tauffenberger, Alexandra Vaccaro, Anais Aulas, Christine Vande Velde, J Alex Parker

**Affiliations:** 1 CRCHUM, Université de MontréalMontréal, Québec, Canada; 2 Centre of Excellence in Neuromics, Université de MontréalMontréal, Québec, Canada; 3 Département de Pathologie et Biologie Cellulaire, Université de MontréalMontréal, Québec, Canada; 4 Département de Médecine, Université de MontréalMontréal, Québec, Canada

**Keywords:** aging, *Caenorhabditis elegans*, metabolism, neurodegeneration, protein folding, proteotoxicity

## Abstract

Nutrient availability influences an organism’s life history with profound effects on metabolism and lifespan. The association between a healthy lifespan and metabolism is incompletely understood, but a central factor is glucose metabolism. Although glucose is an important cellular energy source, glucose restriction is associated with extended lifespan in simple animals and a reduced incidence of age-dependent pathologies in humans. We report here that glucose enrichment delays mutant polyglutamine, TDP-43, FUS, and amyloid-β toxicity in *Caenorhabditis elegans* models of neurodegeneration by reducing protein misfolding. Dysregulated metabolism is common to neurodegeneration and we show that glucose enrichment is broadly protective against proteotoxicity.

## Introduction

The accumulation of misfolded proteins in the nervous system is a hallmark of many late-onset neurodegenerative diseases. Cellular mechanisms to combat proteotoxicity decline with age, suggesting that aging may directly impact neurodegenerative disease onset and progression. Proteotoxicity is associated with a number of phenotypes including oxidative stress, transcriptional and metabolic disturbances. Consistently, a number of mechanisms linked to aging and neuroprotection include genes and pathways that regulate metabolism and energy production including dietary restriction (DR; Kenyon, 2005).

Glucose is a major energy molecule whose levels are actively regulated. Disrupted glucose homeostasis can lead to obesity, type 2 diabetes, and cardiovascular diseases in humans (Venn & Green, 2007). Consistently, in simple models like worms, both DR and glucose restriction slow aging, while exposure to high glucose shortens lifespan (Schulz et al., 2007; Lee et al., 2009). Furthermore, in many systems, DR improves glucose homeostasis and ameliorates insulin insensitivity with concomitant health improvements. However, glucose is the main energy source in human neurons and the brain is estimated to consume over 50% of total glucose in the body (Fehm *et al.*, 2006). Neurons have limited energy storage capacity despite their high-energy demands and are susceptible to energy fluctuations, and glucose homeostasis abnormalities are observed in amyotrophic lateral sclerosis (ALS) and Huntington’s disease (Podolsky & Leopold, 1977; Lalic *et al.*, 2008; Pradat *et al.*, 2010). We set out to examine the contribution of either DR or elevated glucose levels in *Caenorhabditis elegans* proteotoxicity models.

## Results

### Caloric restriction is ineffective against neuronal proteotoxicity

**Fig. 1:**
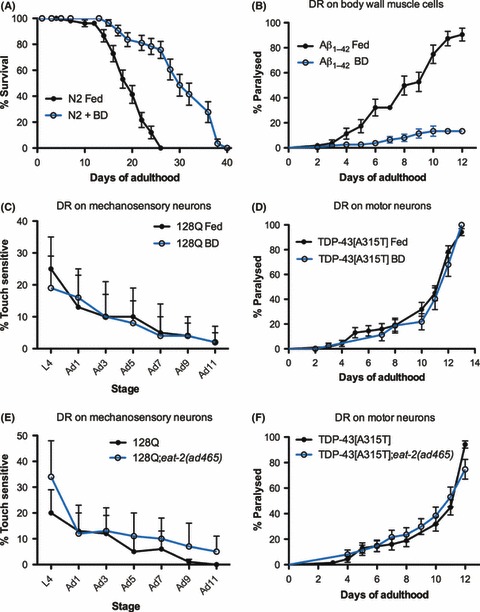
Dietary restriction does not reduce neuronal proteotoxicity. (A) Bacterial deprivation (BD) of adult animals increased the lifespan compared to normally fed (*ad libitum*) wild-type N2 animals. (B) BD delayed the paralysis phenotype caused by the expression of Aβ_1-42_ in worm body wall muscles compared to *ad libitum* transgenics (*P* < 0.001). (C) BD had no effect on the loss of touch sensitivity induced by the expression of mutant polyglutamine (128Q) in worm mechanosensory neurons compared to *ad libitum* transgenics. (D) The progressive paralysis phenotype caused by the expression of mutant TDP-43 in worm motor neurons was unaffected by BD conditions compared to *ad libitum* transgenics. (E) The *eat-2(ad465)* mutation reduces feeding and induces dietary restriction conditions in worms but had no effect on 128Q toxicity in worm mechanosensory neurons. (F) Mutant TDP-43 toxicity in worm motor neurons was unaffected by the *eat-2(ad465)* mutation.

We investigated DR in worms with two methods: by removing their bacterial food source (BD, bacterial deprivation) and with the genetic surrogate *eat-2* mutation that reduces the worms’ ability to ingest food (Lakowski & Hekimi, 1998; Smith *et al.*, 2008). As previously reported, BD extends the lifespan of wild-type worms (Fig. 1A and Table S1) and suppressed paralysis phenotypes of worms expressing an amyloid-β fragment (Aβ_1-42_) in body wall muscle cells (Fig. 1B; Kaeberlein *et al.*, 2006; Steinkraus *et al.*, 2008). We examined neuronal proteotoxicity with a well-characterized expanded polyglutamine (polyQ, with 128Q) model in *C. elegans* touch receptor neurons (Parker *et al.*, 2001, 2005) and with a worm strain that expresses full length human mutant TDP-43[A315T] (mTDP-43), a mutation linked to ALS (Kabashi *et al.*, 2008) in *C. elegans* GABAergic motor neurons (Vaccaro *et al.*, 2012). mTDP-43 transgenics show adult-onset motility defects leading to progressive paralysis, aggregation of mTDP-43 in motor neurons, and neurodegeneration with no effects on lifespan (Vaccaro *et al.*, 2012). Neither BD nor *eat-2* reduced polyQ or mTDP-43 toxicity in neurons (Fig. 1C–F) leading us to conclude that DR conditions cannot ameliorate neuronal proteotoxicity in our models. Of note, caloric restriction was recently shown to shorten the lifespan of an ALS mouse model based on the expression of mutant superoxide dismutase (Patel *et al.*, 2010) and was ineffective in reducing neuronal dysfunction in a Drosophila model of amyloid-β toxicity (Kerr *et al.*, 2011).

### Glucose delays proteotoxicity in age-dependent disease models

We next examined conditions opposite to DR, namely high glucose concentrations on proteotoxicity. Using culture conditions shown to have physiological effects (Lee *et al.*, 2009), worms grown from hatching on plates containing 2%d-glucose (GE, glucose enrichment) showed an approximate fourfold increase in internal glucose levels (Fig. S1A). We measured glycolytic flux of GE by examining the levels of pyruvate, which is a downstream intermediate metabolite of glycolysis, and we observed that worms grown on GE conditions had increased internal pyruvate levels (Fig. S1B).

**Fig. 2:**
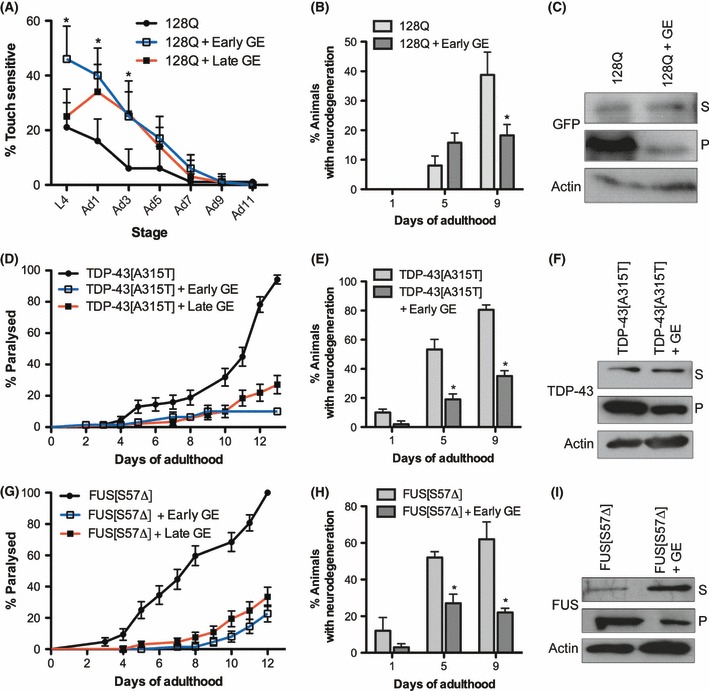
Glucose enrichment (GE) delays proteotoxicity in *Caenorhabditis elegans.* (A) Touch sensitivity was improved by early GE for L4 stage 128Q transgenics and for 128Q animals subjected to either early or late GE at adult days 1-3 relative to untreated 128Q worms (*P < 0.05). (B) GE reduced axonal degeneration phenotypes in adult day 9 128Q animals (*P < 0.05 compared to untreated 128Q). (C) Soluble supernatant (S) and insoluble pellet (P) fractions from 128Q worms grown with or without GE were compared by immunoblotting against GFP. GE reduced the amount of insoluble 128Q::CFP fusion protein levels compared to 128Q worms grown without GE. (D) GE reduced mTDP-43-associated age-dependent paralysis (*P* < 0.001 for GE transgenics vs. untreated transgenics). (E) Early GE reduced mTDP-43-associated age-dependent motor neuron degeneration (*P < 0.001 at days 5 and 9 of adulthood compared to untreated transgenics). (F) GE reduced the amount of insoluble mTDP-43 protein in pellet (P) fractions compared to TDP-43 worms not grown on GE. (G) GE reduced mFUS-associated paralysis (*P* < 0.001 for early or late GE vs. untreated transgenics). (H) Early GE reduced the progressive neuronal degeneration observed in mFUS transgenics (*P < 0.001 at days 5 and 9 compared to untreated transgenics). (I) GE reduced the amount of insoluble mFUS protein in pellet fractions compared to mFUS worms not grown on GE.

We then tested whether GE modulated proteotoxicity in our three different worm models: 128Q in mechanosensory neurons, mTDP-43 in motor neurons, and a second ALS model based on the expression of an ALS-associated FUS mutation (FUS[S57Δ] or mFUS; Belzil *et al.*, 2011) in motor neurons that also show age-dependent paralysis and motor neuron degeneration phenotypes (Vaccaro *et al.*, 2012). We tested transgenic worms grown on glucose from hatching (early GE) as well as animals transferred to 2% glucose plates at the late L4 larval stage (late GE). Treatment with early or late GE improved touch insensitivity in animals expressing 128Q in touch receptor neurons (Fig. 2A). The 128Q strains show age-dependent degeneration of axonal processes along and the formation of insoluble polyQ proteins in protein extracts from whole worms (Parker *et al.*, 2001). GE reduced neuronal degeneration in the 128Q animals, and immunoblot analysis showed a decrease in the amount of insoluble protein from 128Q worm extracts (Fig. 2B,C). Looking at our motor neuron models, we observed that GE reduced the paralysis and neuronal degeneration phenotypes caused by the expression of mutant mTDP-43 or mFUS and reduced the fraction of insoluble mutant proteins (Fig. 1D–I), suggesting that GE may reduce the cellular load of toxic misfolded proteins. GE had no effect on transgenic strains expressing wild-type polyQ, TDP-43, or FUS proteins (Fig. S2). Additionally, GE reduced paralysis phenotypes and the amount of toxic oligomers in transgenic worms expressing Aβ_1-42_ in body wall muscle cells (Fig. S3).

### Neuroprotection by glucose requires glycolysis

**Fig. 3:**
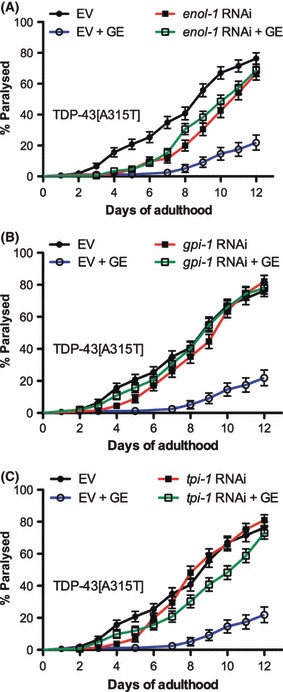
Glycolysis genes are required for glucose neuroprotection. mTDP-43 worms grown in plates with 2% glucose enrichment (GE) and empty vector (EV) RNAi had reduced levels of paralysis compared to untreated EV mTDP-43 worms (*P* < 0.001). RNAi against genes involved in glycolysis including (A) *enol-1*, (B) *gpi-1,* or (C) *tpi-1* all blocked the protective effects of GE compared to EV + GE controls (*P* < 0.001).

To determine whether protection against proteotoxicity required glycolysis, we tested l-glucose, which is an enantiomer of d-glucose that cannot be phosphorylated by hexokinase and thus cannot enter the glycolysis pathway. We observed that 128Q, mTDP-43, and mFUS transgenics grown on 2%l-glucose were indistinguishable from untreated transgenics, demonstrating that d-glucose specifically rescues proteotoxicity (Fig. S4). These data are consistent with a previous study showing l-glucose had no discernable effects in *C. elegans* lifespan studies (Lee *et al.*, 2009). To directly test whether glycolysis was essential for neuroprotection, we focused on three glycolytic enzymes represented by a single member in the *C. elegans* genome including *enol-1* (enolase 1), *gpi-1* (glucose 6-phosphate isomerase), and *tpi-1* (triosephosphate isomerase). In each case, RNAi against these genes completely blocked the rescuing effect of GE against mutant TDP-43 paralysis (Fig. 3). Thus, glucose metabolism is essential for the protective effects of GE against proteotoxicity.

### Glucose reduces lifespan and progeny numbers

The toxic effects of high dietary glucose are well documented and are known to decrease lifespan in worms (Lee *et al.*, 2009; Mondoux *et al.*, 2011). In *C. elegans,* long-lived *daf-2* insulin/IGF receptor mutants and wild-type worms grown from adulthood under GE conditions are reported to have decreased lifespan, but GE did not further reduce the lifespan of the short-lived FOXO transcription factor *daf-16* mutants (Lee *et al.*, 2009). We grew worms under early and late GE conditions and confirmed that both early and late GE reduced the lifespan of *daf-2* mutants (Fig. S5A and Table S1) but did not reduce the lifespan of wild-type N2 worms or *daf-16* mutants (Fig. S5B,C and Table S1). Furthermore, we observed no reduction from GE on the lifespan of 128Q, mTDP-43, mFUS, or Aβ_1-42_ transgenic animals (Fig. S5D–G and Table S1).

**Fig. 4:**
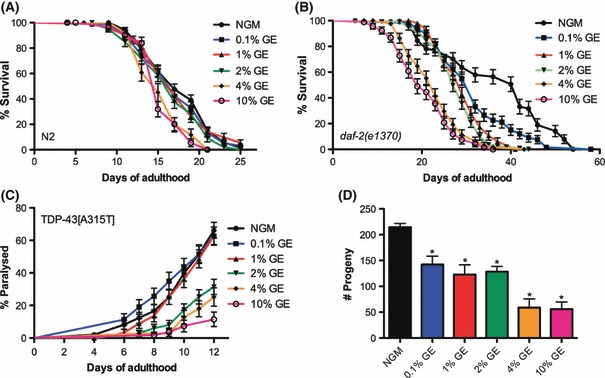
Dose-dependent effects of glucose on lifespan and proteotoxicity. Strains were grown on plates from hatching with either 0% (NGM), 0.1%, 1%, 2%, 4%, or 10% glucose enrichment (GE). (A) Wild-type N2 worms grown on 0.1%, 1%, or 2% GE had lifespans indistinguishable from worms on regular NGM media. N2 worms grown on 4% or 10% had significantly shorter lifespans than worms grown on regular media. (B) *daf-2(e1370)* worms grown on all GE concentrations had significantly decreased lifespans compared to *daf-2* worms grown on normal media. The largest decrease in lifespan was for worms grown on 4% and 10% GE plates. (C) mTDP-43 worms grown on 0.1% or 1% GE had similar rates of paralysis compared to mTDP-43 transgenics grown on normal media. TDP-43 worms grown on 2%, 4%, or 10% had significantly reduced rates of paralysis compared to mTDP-43 worms grown on normal media. (D) Wild-type N2 worms grown on 0.1%, 1%, 2%, 4%, or 10% had reduced brood sizes compared to worms grown on normal NGM media. N2 animals grown on 4% and 10% had the fewest progeny (*P < 0.001 compared to N2 worms grown on NGM).

We further explored glucose’s negative effects on lifespan and protective effects against proteotoxicity by testing different concentrations of glucose in lifespan and proteotoxicity assays. We tested wild-type N2 worms and long-lived *daf-2* mutants grown from hatching on plates with different concentrations of glucose including no glucose, 0.1%, 1%, 2%, 4%, and 10% GE. In N2 worms, we observed no negative effects of GE on lifespan at concentrations of 0.1%, 1%, or 2%, while significant decreases in lifespan were observed for 4% and 10% GE (Fig. 4A). For *daf-2* worms, we observed that all concentrations of GE reduced lifespan with the most severe effects noted for 4% and 10% GE (Fig. 4B). Thus, GE has a dose-dependent effect on lifespan but there may also be a contribution from the duration of exposure; the apparent negative effects of 0.1%, 1%, and 2% GE in *daf-2* worms may not have time to manifest in the shorter lived N2 strain. Looking at the GE concentrations and proteotoxicity, we observed that 0.1% and 1% GE had no effect on the rate of paralysis for mTDP-43 worms, while 2%, 4%, and 10% GE all significantly reduced mTDP-43 toxicity (Fig. 4C). Finally, glucose is reported to reduce progeny numbers of wild-type worms (Lee *et al.*, 2009; Mondoux *et al.*, 2011), and we observed all concentrations of glucose reduced brood size with the greatest effects for 4% and 10% GE (Fig. 4D). These data suggest glucose has dose-dependent effects and that the protective effects of GE against proteotoxicity can be uncoupled from the negative effects on lifespan and reproduction.

### Glycerol does not reduce neuronal dysfunction

Besides its role as an energy source in glycolysis, glucose can be metabolized to glycerol, and increased glycerol levels are believed to prevent the aggregation of proteins damaged during hypertonic stress (Lamitina *et al.*, 2004; Choe & Strange, 2008). We observed that N2 worms exposed to GE had increased concentrations of internal glycerol, as did worms exposed to glycerol on plates (Fig. S6A,B). To directly test whether glycerol was responsible for neuroprotection, we grew mTDP-43 worms on plates supplemented with 2% glycerol but saw no reduction in paralysis compared to untreated controls (Fig. S6C). Thus, we conclude the neuroprotection observed from GE is not because of a concomitant increase in internal glycerol levels.

### Additional sugars reduce proteotoxicity

To learn whether neuroprotection was limited to glucose, we examined several other sugars including sucrose, galactose, and fructose. We observed that all three sugars reduced paralysis phenotypes of mTDP-43 worms although galactose was the least effective (Fig. S7A). Next, we looked at lifespan and consistent with what we observed for glucose all three sugars reduced the lifespan of wild-type N2 worms (Fig. S7B). Thus, the neuroprotective and lifespan-limiting phenotypes we observe may be a feature common to many sugars.

### Glucose restores protein homeostasis and protects against environmental stress

**Fig. 5:**
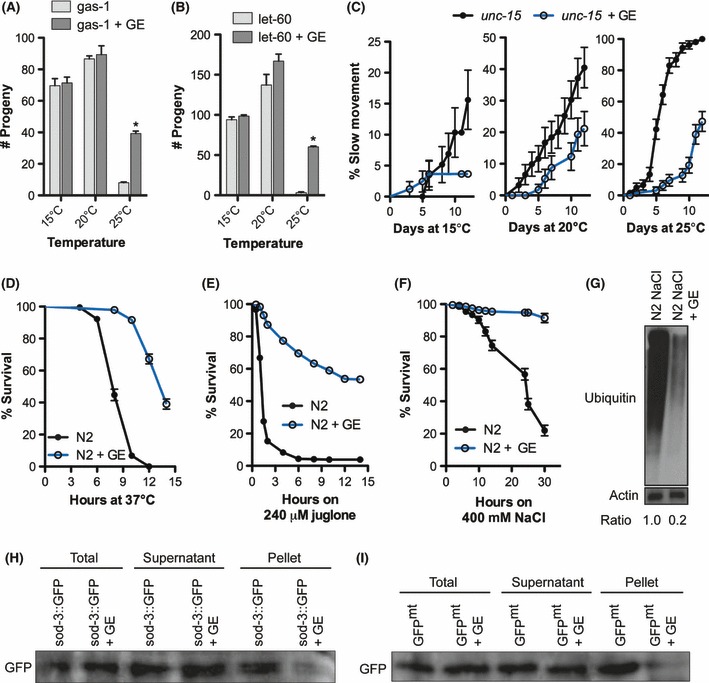
Glucose protects against protein misfolding and environmental stress. (A) GE rescued the low-progeny phenotype of the temperature-sensitive *gas-1* mutant at the restrictive temperature of 25 °C (*P < 0.001 vs. untreated *gas-1* mutants). (B) GE rescued the low-progeny phenotype of the temperature-sensitive *let-60* mutant at the restrictive temperature of 25 °C (*P < 0.001 vs. untreated *let-60* mutants). (C) GE reduced the progressive, age-dependent slow movement phenotype of *unc-15* mutants at 20 °C (*P* < 0.05 vs. untreated *unc-15* mutants) and 25 °C (*P* < 0.001 vs. untreated *unc-15* mutants). (D) N2 worms grown on GE plates were resistant to thermal stress compared to untreated controls (*P* < 0.001). (E) Wild-type N2 worms grown on GE plates were resistant to juglone-induced mortality compared to untreated controls (*P* < 0.001). (F) Glucose protected wild-type N2 worms against high NaCl toxicity (*P* < 0.001) compared to untreated controls. (G) Representative Western blot and quantification of high molecular weight ubiquitin conjugates of worms exposed to NaCl or grown on GE plates before exposure to NaCl stress. Treatment with GE reduced the amount of ubiquitinylation levels caused by hypertonic stress. (H) Representative Western blot of protein extracts (total, supernatant, or pellet) from SOD-3::GFP transgenic worms exposed to tunicamycin, or tunicamycin with 2% GE. The amount of insoluble GFP in the pellet fraction was reduced in animals grown under GE conditions. (I) Representative Western blot of protein extracts (total, supernatant, or pellet) from GFP^mt^ transgenic worms exposed to ethidium bromide ± 2% GE. The amount of insoluble GFP in the pellet fraction was greatly reduced in animals grown under GE conditions.

We further explored the notion that glucose reduces protein misfolding with a number of misfolding sensors by using worms with temperature-sensitive (*ts*) destabilizing mutations including *gas-1*, a mitochondrial complex I subunit, *let-60*/RAS, *unc-15*/paramyosin, and *unc-54*/myosin. These animals are viable at the permissive temperature (15 °C) but their gene products are impaired at restrictive temperatures (20 °C or higher) and produce a range of phenotypes including sterility, lethality, or impaired movement (Gidalevitz *et al.*, 2006). When these *ts* mutants were grown on GE plates from hatching and shifted to non-permissive temperatures as adults and scored, we observed that GE rescued many *ts* phenotypes. Sterility for *gas-1* and *let-60* mutants at 25 °C was rescued by GE (Fig. 5A,B). Furthermore, GE rescued the slow movement phenotype of *unc-15* (Fig. 5C) and *unc-54* (Fig. S8) mutants at all temperatures tested. These data suggest that glucose has a previously unrecognized ability to counteract protein misfolding.

Looking beyond genetically encoded proteotoxicity and given the links between metabolism, longevity, and the cellular stress response, we wondered whether GE protected against environmentally induced protein damage. Elevated temperatures can cause widespread protein damage within the cell (Morimoto, 2008), and we observed that worms subjected to GE from hatching were more resistant to heat-induced mortality than worms maintained on normal plates (Fig. 5D). Juglone is a natural compound from the black walnut tree that increases intracellular concentrations of superoxide resulting in oxidative stress and covalent damage to proteins (Van Raamsdonk & Hekimi, 2009; Ferguson *et al.*, 2010). Juglone causes near complete mortality of wild-type worms by 14 hours in our assay, and wild-type N2 worms subjected to GE from hatching and transferred to juglone plates as young adults were highly resistant to juglone-induced mortality (Fig. 5E). GE also protected wild-type worms against hypertonic stress (Fig. 5F). Hypertonic stress from high NaCl concentrations is known to cause extensive protein damage that can be assayed by staining for ubiquitin conjugation (Choe & Strange, 2008). GE greatly reduced the amount of high molecular weight ubiquitin conjugates compared to worms grown on NaCl alone (Fig. 5G). These data suggest that glucose protects proteins against misfolding and damage from multiple environmental stresses.

We next directly tested whether GE could reduce the amount of misfolded proteins. Worm strains that express GFP in the cytoplasm (*sod-3p::GFP*) or in the mitochondria (*myo-3p::GFP*^*mt*^) were subjected to stress resulting in compartmental specific protein misfolding (Yoneda *et al.*, 2004). Tunicamycin is a compound that interferes with the endoplasmic reticulum (ER)-specific N-linked glycosylation leading to ER stress and exacerbates protein misfolding (Yoneda *et al.*, 2004). Immunoblot analysis of *sod-3p::GFP* worms grown on tunicamycin plates showed a large amount of the GFP signal resided in the pellet or insoluble fraction indicating protein misfolding had occurred and that treatment with GE greatly reduced the GFP signal in the insoluble fractions (Fig. 5H). Similarly, ethidium bromide disrupts protein processing in the mitochondria leading to increased protein misfolding (Yoneda *et al.*, 2004). Immunoblotting from *myo-3p::GFP*^*mt*^ worms grown on ethidium bromide plates showed a large GFP signal in the insoluble fraction and that treatment with GE reduced the amount of insoluble proteins (Fig. 5I). These data show that GE has a cellular-wide ability to counteract protein misfolding and reduce the amount of insoluble proteins.

### Neuroprotection by glucose requires DAF-16 and HSF-1

**Fig. 6:**
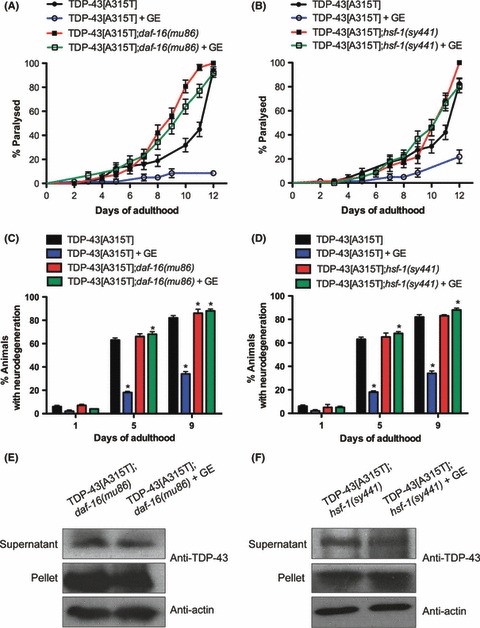
Glucose neuroprotection is *daf-16* and *hsf-1* dependant. (A) GE rescued mTDP-43 paralysis (*P* < 0.001 vs. non-treated mTDP-43 worms). *daf-16(mu86)* enhanced paralysis (*P* < 0.001 vs. mTDP-43 alone) and abolished the protective effect of GE against mTDP-43-associated paralysis in transgenic worms [*P* < 0.001 mTDP-43;*daf-16(mu86)* + GE vs. mTDP-43 + GE]. (B) GE reduced mTDP-43 paralysis (*P* < 0.001 vs. non-treated mTDP-43 worms) and this was dependent on *hsf-1* [*P* < 0.001 mTDP-43;*hsf-1(sy441)* + GE vs. mTDP-43 + GE]. (C) GE suppressed motor neuron degeneration at adult days 5 and 9 in worms expressing mTDP-43, but this protective effect was lost in animals mutant for *daf-16* or (D) *hsf-1* (*P < 0.001 vs. mTDP-43 alone). mTDP-43 proteins remained highly insoluble after glucose treatment in (E) *daf-16* and (F) *hsf-1* mutants.

The accumulation of misfolded proteins is a hallmark of neurodegeneration and a natural consequence of aging (David *et al.*, 2010). Among other mechanisms, cells utilize chaperone networks to maintain protein homeostasis and central to this are the Insulin/IGF and heat shock factor 1 (HSF-1) pathways (Morimoto, 2008). In worms, downstream from insulin/IGF signaling via *daf-2* are *daf-16* and *hsf-1*, both of which have been shown to modulate the aggregation and toxicity of misfolded proteins (Morley *et al.*, 2002; Hsu *et al.*, 2003; Parker *et al.*, 2005; Cohen *et al.*, 2006; Zhang *et al.*, 2011). To directly test their role in glucose-mediated neuroprotection, we crossed our mTDP-43 transgenics with the loss of function mutants *daf-16(mu86)* and *hsf-1(sy441)*. Mutation in either gene abolished the protective effects of glucose compared to controls (Fig. 6A,B). Mutation in *daf-16* or *hsf-1* also blocked the protective activity of glucose against motor neuron degeneration (Fig. 6C,D) and suppressed glucose’s capacity to reduce the levels of insoluble TDP-43 mutant proteins (Fig. 6E,F). These data suggest that GE requires the protein homeostasis activities of *daf-16* and *hsf-1* to protect neurons against protein misfolding. Multiple glucose concentrations were tested but none of them further reduced the lifespan of *daf-16* and *hsf-1* mutants suggesting these genes are downstream effectors of glucose activity (Fig. S9A,B). Using N2 worms, we tested whether GE affected transcription of *daf-16* and *hsf-1*, plus a number of *daf-16* and *hsf-1* transcriptional targets, but observed no difference between GE and non-treated controls (Fig. S9C–E). These data suggest that GE does not induce the transcription of protein quality control genes but may generally require a functional protein homeostasis network for its neuroprotective properties. To confirm this idea, we examined whether deletion of genes required for additional protein homeostasis mechanisms were required for protection against mTDP-43 paralysis. *ire-1* encodes the *C. elegans* orthologue of the ER transmembrane protein inositol-requiring kinase 1 (IRE1) and is essential for a branch of the ER unfolded protein response (Calfon *et al.*, 2002). Mutation of *ire-1* blocks the rescuing effects of glucose against mTDP-43 (Fig. S10A). WWP-1 is an E3 ubiquitin ligase important for ubiquitination and proteasomal degradation of proteins (Carrano *et al.*, 2009), and mutation in *wwp-1* also blocks the protective effects of glucose against mutant TDP-43 (Fig. S10B). Thus, GE likely employs multiple protein homeostasis mechanisms to reduce the toxicity of misfolded proteins.

## Discussion

Model organisms are providing insights into the links between metabolism, longevity, and age-dependent pathologies. DR is a well-studied lifespan-enhancing phenomenon under examination for its potential to delay age-dependent afflictions. However, DR failed to ameliorate neuronal phenotypes in our polyQ and ALS models leading us to question the role of DR mimetics as therapeutics for neurodegeneration. A major mitigating factor may be the relative effects of DR in different tissues; we observed no benefit from DR in neuronal proteotoxicity models, but we and others have observed the protective effects of DR against proteotoxicity in muscle-based expression systems (Steinkraus *et al.*, 2008). Indeed, there is evidence that *C. elegans* muscles and neurons are not equivalent in their chaperone activity in handling protein misfolding during aging (Kern *et al.*, 2010).

The inability of DR to delay neuronal proteotoxicity in our systems led us to investigate glucose metabolism. Our work identifies a new role for the ubiquitous energy molecule glucose in protein folding and neuroprotection. Glucose metabolism is central to biology, and the maintenance of glucose homeostasis has profound effects on development, health, fertility, and lifespan of many organisms. Work with simple models like *C. elegans* is beginning to shed light on conserved aspects of excess glucose intake, or the glucose stress response (Schulz *et al.*, 2007; Lee *et al.*, 2009; Mondoux *et al.*, 2011). In worms, elevated glucose intake has pleiotropic effects with negative impacts on lifespan, fertility, and dauer formation (Lee *et al.*, 2009; Mondoux *et al.*, 2011). In humans, excessive intake of glucose and other sugars is associated with a number of health problems including obesity, type II diabetes, and neuronal toxicity (Giacco & Brownlee, 2010). Importantly, our data demonstrate that the negative effects on lifespan from excessive glucose can be separated from neuroprotection. We clearly show dose-dependent effects where neurons expressing toxic proteins respond favorably to glucose even though high glucose concentrations lead to decreased lifespan among other outcomes.

Our data suggest that glucose achieves neuroprotection by reducing the levels of misfolded mutant proteins within the cell. This activity requires the network of chaperone proteins under control of the stress and cellular survival genes *daf-16* and *hsf-1* because deletion of either of these genes blocked glucose’s neuroprotective effects. These findings are consistent with the known role of these genes in protecting against neuronal proteotoxicity (Parker *et al.*, 2005; Zhang *et al.*, 2011). Even though *daf-16* and *hsf-1* are required for neuroprotection, GE does not increase the expression of these genes. Furthermore, decreased lifespan from GE is dependent on *daf-16* and *hsf-1* because lifespan is not further reduced in these mutants consistent with previous findings (Lee *et al.*, 2009).

In simple systems like worms mutations that extend lifespan are often associated with increased cellular stress resistance (Kenyon, 2010). A consequence of these life-extending mutations is often extensive metabolic reprogramming (Artal-Sanz & Tavernarakis, 2008), and our work suggests that certain metabolic outcomes are more favorable to neurons expressing toxic proteins than others. Future work carefully examining the metabolic profiles of diseased and aging neurons may help delineate pathogenic mechanisms and potential therapeutic approaches. In conclusion, we describe an unexpected role for glucose in cellular protection, and strategies to supplement neuronal glucose levels may reduce neuronal proteotoxicity.

## Materials and methods

### Worm strains and genetics

Standard methods of culturing and handling worms were used. Worms were maintained on standard NGM plates streaked with OP50 *Escherichia coli.* In some experiments, d-glucose or l-glucose was added to NGM plates (All products from Sigma, St. Louis, MO, USA). All strains were scored at 20 °C unless indicated. Mutations and transgenes used in this study were *daf-2(e1370), daf-16(mu86), dvIs2[unc-54::Aβ*_*1-42*_*;rol-6(su1006*)], *eat-2(ad465), gas-1(fc21), igIs1[mec-3::htt57Q128::CFP;mec-7::YFP;lin-15(+)], igIs245[mec-3::htt57Q19::CFP;mec-7::YFP;lin-15(+)], let-60(ga89), muIs84[sod-3::GFP], unc-15(e1402), unc-54(e1301), zcIs14[myo-3::GFP(mit)], xqIs133[unc-47::TDP-43[A315T];unc-119(+)],* and *xqIs98[unc-47::FUS[S57Δ].* Most of the strains were obtained from the *C. elegans* Genetics Center (University of Minnesota, Minneapolis, MN, USA). Mutants or transgenic worms were verified by visible phenotypes, PCR analysis for deletion mutants, sequencing for point mutations, or a combination thereof. Deletion mutants were out-crossed a minimum of three times to wild-type worms prior to use.

### Worm behavioral tests

Touch tests were conducted and scored as previously described (Parker *et al.*, 2007). Briefly, for late GE experiments, 128Q animals were transferred to glucose plates at the L4 stage, while early GE animals were exposed to glucose from hatching. The animals were assayed for touch responsiveness at the L4 stage and subsequently tested at adult days 1–11. For worms expressing Aβ_1-42_, mutant TDP-43 or FUS animals were counted as paralyzed if they failed to move upon prodding with a worm pick. Worms were scored as dead if they failed to move their head after being prodded in the nose and showed no pharyngeal pumping. For the paralysis tests, worms grown on glucose from hatching were transferred to the appropriate experimental plate for scoring.

### Fluorescence microscopy

For scoring of neuronal processes from 128Q, mTDP-43, and mFUS transgenics, animals were selected at days 1, 5, and 9 of adulthood for visualization of mechanosensory (128Q) or motor neurons (mTDP-43 and mFUS) processes *in vivo*. Animals were immobilized in M9 with 5 mm levamisole and mounted on slides with 2% agarose pads. Neurons were visualized with a Leica 6000 and a Leica DFC 480 camera. A minimum of 100 animals were scored per treatment over 4–6 trials. The mean and SEM were calculated for each trial, and two-tailed *t*-tests were used for statistical analysis.

### Measurement of glucose, glycerol, and pyruvate

Extracts from synchronized worms were done as described previously (Lamitina *et al.*, 2004; Lee *et al.*, 2009). Glucose levels were quantified with the Amplex Red Glucose/Glucose Oxidase kit (Molecular Probes-Invitrogen, Life Technologies Carlsbad, CA, USA). Glycerol levels were quantified with Glycerol Free Reagent (Sigma). Pyruvate levels were quantified with a Pyruvate assay kit (Biovision, Milpitas, CA, USA). Protein levels were quantified with a BCA assay kit and used for normalization of glucose or pyruvate levels. The mean and SEM were calculated for each trial, and two-tailed *t*-tests were used for statistical analysis.

### Stress assays

For oxidative stress tests, worms were grown on NGM or NGM + 2% glucose and transferred to NGM plates + 240 μm juglone at adult day 1. For thermal resistance, worms were grown on NGM or NGM 2% glucose and put at 37 °C as adult day 1. For osmotic resistance, worms were grown on NGM or NGM 2% glucose and put on 400 mm NaCl plates at adult day 1. For all assays, worms were evaluated for survival every 30 min for the first 2 h and every 2 h after up to 14 h. Nematodes were scored as dead if they were unable to move in response to heat or tactile stimuli. For all tests worms, 20 animals/plate by triplicates were scored.

### Lifespan assays

Worms were grown on NGM or NGM + glucose and transferred on NGM-FUDR or NGM-FUDR + glucose. Twenty animals/plate by triplicates were tested at 20 °C from adult day 1 until death. Worms were declared dead if they did not respond to tactile or heat stimulus. For BD experiments, hypochlorite-extracted N2 worms were grown on UV-killed OP50 bacteria. LB cultures were streaked with UV-killed bacteria and grown overnight to confirm their inability to grow. At day 1 of adulthood, N2 worms were transferred to NGM plates without bacteria. Worms were transferred to new NGM plates without bacteria in a flow hood to avoid bacterial contamination that could act as a food source.

### RNAi paralysis tests

RNAi-treated strains were fed with *E. coli* (HT115) containing an Empty Vector (EV), *enol-1*(T21B10.2)*, tpi-1*(Y17G7B.7), *and gpi-1*(Y87G2A.8) RNAi clones from the ORFeome RNAi library. RNAi experiments were performed at 20 °C. Worms were grown on either NGM or NGM + 2% glucose both enriched with 1 mm isopropylb-d-thiogalactopyranoside. Worms were scored for paralysis as described before from adult days 1 to 12.

### Progeny tests (glucose dosage)

For scoring progeny under different glucose conditions, 10 L4 worms were grown on NGM (0% glucose) plates enriched with either 0.1%, 1%, 2%, 4%, or 10% glucose and placed at 20 °C. Over the next 2 days, individual worms were transferred on new plates, and L1 larvae were scored for each plate.

### Bacterial deprivation experiments

Following a previously described protocol (Kaeberlein *et al.*, 2006), hypochlorite-extracted worm population was grown on a UV-killed bacteria to avoid any contamination. Worms were transferred to NGM plates with no bacteria and tested from adult days 1 to 12. Nematodes were moved to a new empty plate every day to avoid any bacterial contamination that could act as a food source. Worms were scored as paralyzed if they were not able to move in response to tactile stimulus and as dead if they were not able to move their head after tactile stimulus. All the experiment were run at 20 °C, 20 worms/plate by triplicates. Touch tests were carried out as described above.

### Temperature-sensitive strains experiments

For scoring *gas-1(fc21)* and *let-60(ga89)* progeny, five synchronized L4 worms were grown on NGM plates with or without 2% glucose (1 animal/plate) and were placed at 15, 20, or 25 °C overnight to lay eggs. The next 2 days, the worms were individually transferred to new plates: L1 worms were scored for each plate. For the *unc-15(e1402)* and *unc-54(e1301)* slow movement phenotypes, 60 synchronized L4 animals were grown from hatching on NGM plates with or without 2% glucose (20 animals/plate) and were placed at 20 °C. Over 12 days, worms were transferred to a new plate everyday, placed into a 1-cm-diameter circle and after 5 min animals that did not move from the circle were scored. The mean and SEM were calculated for each trial, and two-tailed *t*-tests were used for statistical analysis.

### Protein ubiquitination after NaCl treatment

N2 worms were grown on NGM or NGM + 2% glucose plates, washed with M9, and grown for 24 h on 400 mm NaCl plates. The next day, worms were collected with M9, and pellets were made for Western blotting.

### GFP solubility experiments

>For *sod-3::GFP*, worms were grown on NGM or NGM + 2% glucose plates, washed with M9, and grown for 24 h on 5 mg/mL tunicamycin plates. The next day, worms were collected with M9 and pellets were made for Western blotting. For *myo-3::GFP*^*mt*^, worms were grown on NGM or NGM + 2% glucose plates, washed with M9, and grown for 24 h on 125 mg/mL ethidium bromide plates. The next day, worms were collected with M9, and pellets were made for Western blotting.

### Worm lysates

Worms were collected in M9 buffer, washed 3 times with M9, and pellets were placed at −80 °C overnight. Pellets were lysed in RIPA buffer (150 mm NaCl, 50 mm Tris pH 7.4, 1% Triton X-100, 0.1% SDS, 1% sodium deoxycholate) + 0.1% protease inhibitors (10 mg/mL leupeptin, 10 mg/mL pepstatin A, 10 mg/mL chymostatin LPC;1/1000). Pellets were passed through a 27_1/2_ G syringe 10 times, sonicated, and centrifuged at 16 000 *g*. Supernatants were collected.

For 128Q, TDP-43, and FUS transgenics soluble/insoluble fractions, worms were lysed in extraction buffer [1 m Tris–HCl pH 8, 0.5 m EDTA, 1 m NaCl, 10% NP40 + protease inhibitors (LPC;1/1000)]. Pellets were passed through a 27_1/2_ G syringe 10 times, sonicated, and centrifuged at 100 000 *g* for 5 min. The supernatant was saved as soluble (‘S’) fraction, and the remaining pellet was resuspended in extraction buffer, sonicated, and centrifuged at 100 000 *g* for 5 min. The remaining pellet was resuspended into 100 μL of RIPA buffer, sonicated until the pellet was resuspended in solution, and saved as sample pellet (‘P’). For *sod-3::GFP* and *myo-3::GFP*^*mt*^ soluble/insoluble fractions, worms were lysed in extraction buffer [1 m Tris–HCl pH 8, 0.5 m EDTA, 1 m NaCl, 10% NP40 + protease inhibitors (LPC;1/1000)]. Pellets were passed through a 27_1/2_ G syringe 10 times, sonicated, and centrifuged 16 000 *g*. The supernatant was saved as ‘total protein extract,’ and the remaining pellet was resuspended in extraction buffer, sonicated, and centrifuged at 100 000 *g* for 5 min. The supernatant was saved as ‘S’ fraction, and the remaining pellet was resuspended in extraction buffer, sonicated, and centrifuged at 100 000 *g* for 5 min. The remaining pellet was resuspended into 100 μL of RIPA buffer, sonicated until the pellet was resuspended in solution, and saved as sample ‘P’.

### Protein quantification

All supernatants were quantified with the BCA Protein Assay Kit (Thermo Scientific, Waltham, MA, USA) following the manufacturer instructions.

### Immunoblot

Worm RIPA samples (175 μg/well) were resuspended directly in 1× Laemmli sample buffer, migrated in 12.5% or 10% polyacrylamide gels, transferred to nitrocellulose membranes (BioRad, Hercules, CA, USA), and immunoblotted. Antibodies used were rabbit anti-GFP(1:1000, ab6556; AbCam, Cambridge, UK), rabbit anti-TDP-43 (1:200; Proteintech, Chicago, IL, USA), rabbit anti-FUS/TLS (1:200; AbCam), mouse anti-ubiquitin (1:500; BD, Franklin Lakes, NJ, USA), mouse anti-amyloid Beta 6E10 (1:1000; Calbiochem, Merck, Darmstadt, Germany), and mouse anti-actin (1:10 000; MP Biomedicals, Solon, OH, USA). Blots were visualized with peroxidase-conjugated secondary antibodies and ECL Western Blotting Substrate (Thermo Scientific). Densitometry was performed with Photoshop (Adobe, San Jose, CA, USA).

### Statistics

Statistics of nematode touch-test data were performed using one-way anova, with correction for multiple testing by Tukey–Kramer’s multiple comparison test. Data were expressed as mean ± SD for > 100 nematodes in each group. All experiments were repeated at least three times. *P* < 0.05 was considered significant. For paralysis and stress-resistance tests, survival curves were generated and compared using the Log-rank (Mantel-Cox) test, and a 60–100 animals were tested per genotype and repeated at least three times.

## Supporting Information

**Fig. S1** Worms grown on glucose plates have elevated levels of internal glucose and pyruvate.

**Fig. S2** Glucose has no effect on wild type polyglutamine, TDP-43 or FUS strains.

**Fig. S3** Glucose reduces amyloid-β toxicity.

**Fig. S4** l-glucose does not reduce neuronalproteotoxicity in *C. elegans* transgenics.

**Fig. S5** GE reduces lifespan of long-lived *daf-2* mutants but not strains with shorter lifespans.

**Fig. S6** Glycerol does not reduce mutant TDP-43 toxicity.

**Fig. S7** Other sugars reduce proteotoxicity and lifespan.

**Fig. S8** Glucose rescues *unc-54* mutant phenotypes.

**Fig. S9** Glucose does not affect *daf-16, hsf-1* or target gene expression.

**Fig. S10** Glucose neuroprotection requires protein homeostasis genes.

**Table S1** Lifespan analysis for all experiments.

As a service to our authors and readers, this journal provides supporting information supplied by the authors. Such materials are peer-reviewed and may be re-organized for online delivery, but are not copy-edited or typeset. Technical support issues arising from supporting information (other than missing files) should be addressed to the authors.
