# Supplementary material for: Glucose delays age-dependent proteotoxicity
Source: Aging Cell. 2012 Oct;11(5):856–66. doi: 10.1111/j.1474-9726.2012.00855.x (PMC3470697; doi:10.1111/j.1474-9726.2012.00855.x)
Supplement: Supplementary file 1 [file acel0011-0856-SD1.docx]

Supplementary Information for

**Glucose delays age-dependent proteotoxicity**

Arnaud Tauffenberger^1,2,3,4^, Alexandra Vaccaro^1,2,3,4^, Anais Aulas^1,2,5^, Christine Vande Velde^1,2,5^, and J. Alex Parker^1,2,3,6^

1. CHUM Research Centre (CRCHUM), Montreal, QC, CANADA

2. Centre of Excellence in Neuromics, Universite de Montreal, Montreal, QC, CANADA

3. Département de pathologie et biologie cellulaire, Université de Montréal, Montréal, QC, Canada

4. These authors contributed equally

5. Département de médecine, Université de Montréal, Montréal, QC, Canada

6. Correspondence should be addressed to: ja.parker@umontreal.ca

**
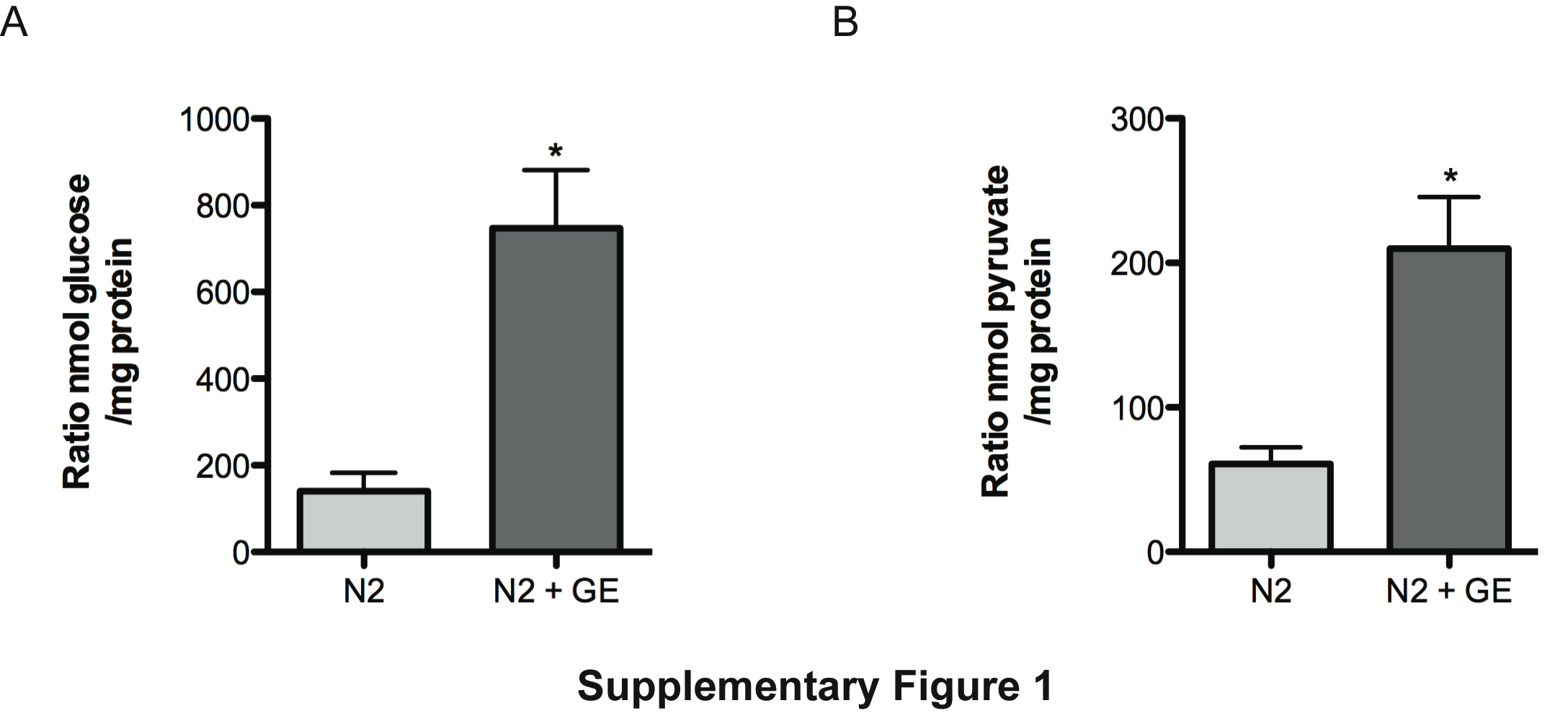
**

**Supplementary Figure 1.** Worms grown on glucose plates have elevated levels of internal glucose and pyruvate.

Worms were grown on 2% glucose plates from hatching, harvested as young adults and assayed for glucose and pyruvate levels. (A) GE increased internal glucose levels (*P< 0.001 versus untreated). (B) GE increased internal pyruvate levels (*P<0.001 versus untreated).


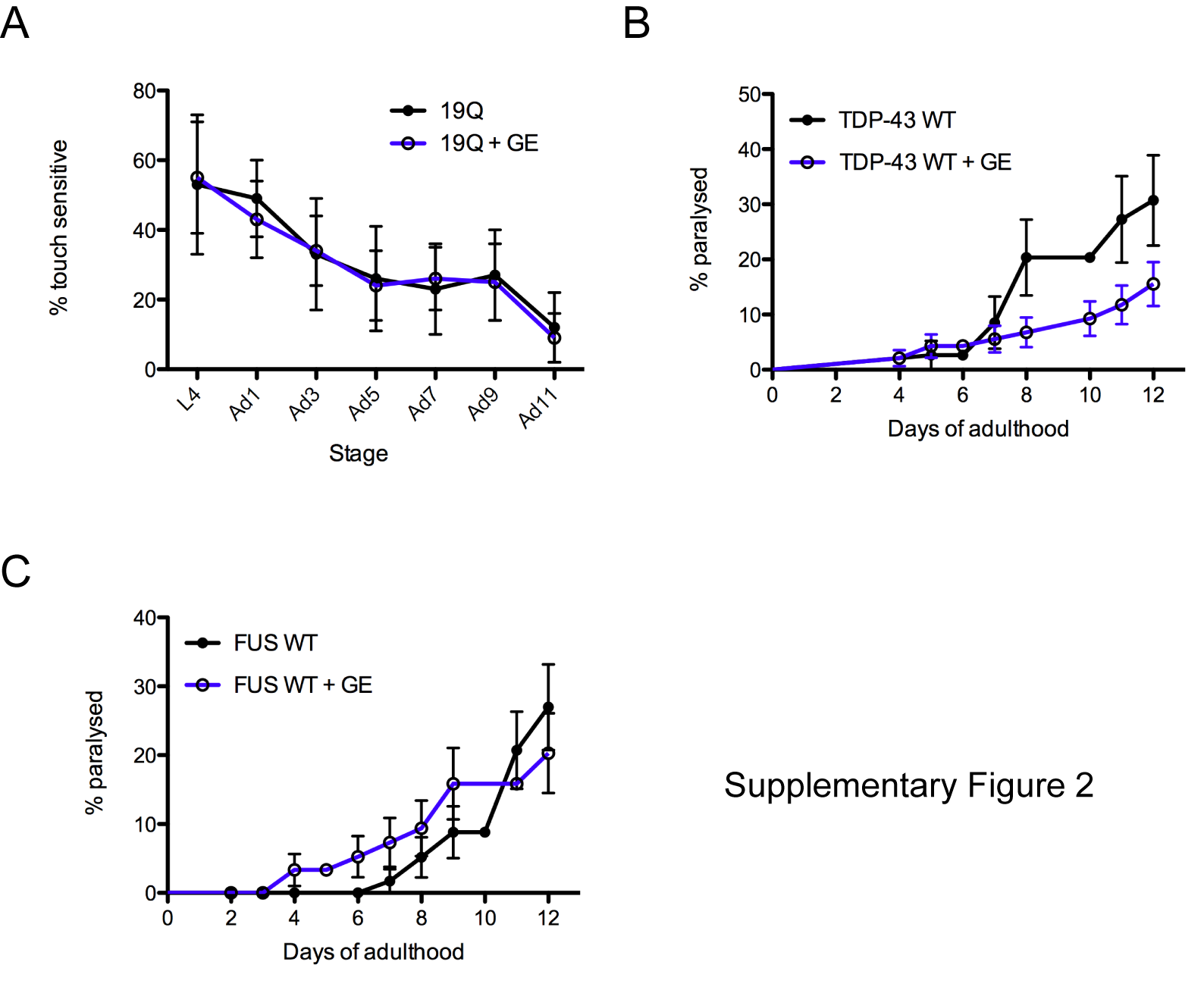


**Supplementary Figure 2.** Glucose has no effect on wild type polyglutamine, TDP-43 or FUS strains.

Treatment with glucose from hatching (early GE) had no effect on (A) the touch responsiveness of animals expressing 19Q in mechanosensory neurons or the rates of paralysis for strains expressing (B) wild type TDP-43 or (C) wild type FUS in motor neurons.


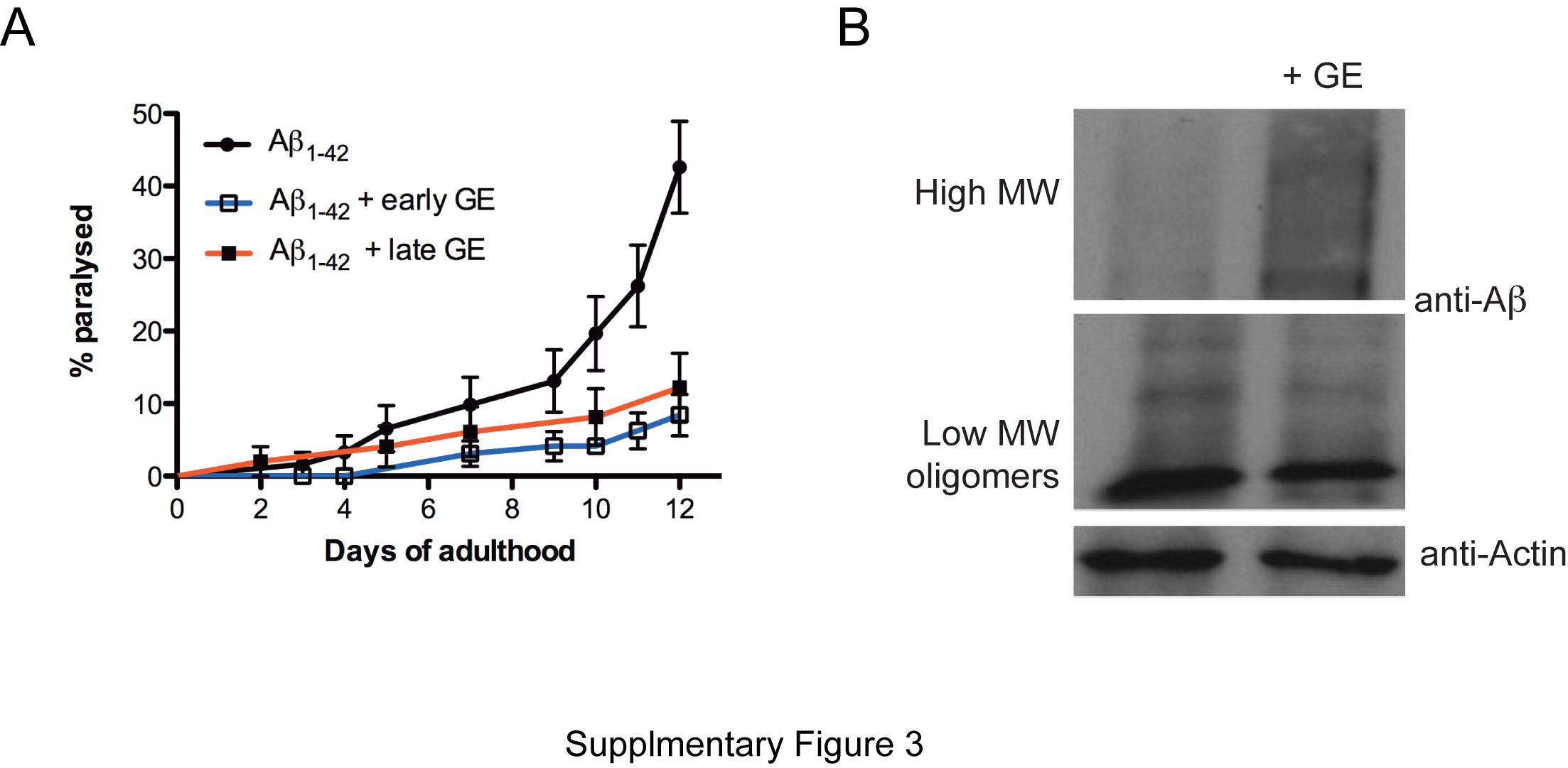


**Supplementary Figure 3.** Glucose reduces amyloid-β toxicity.

(A) Aβ__ worms grown on either early or late GE plates had significantly reduced rates of paralysis compared to untreated control transgenics (P<0.001).  Aβ__ worms exposed to GE showed a reduction in the amount of toxic low molecular weight (MW) oligomers and an increase in the amount of protective high MW species.


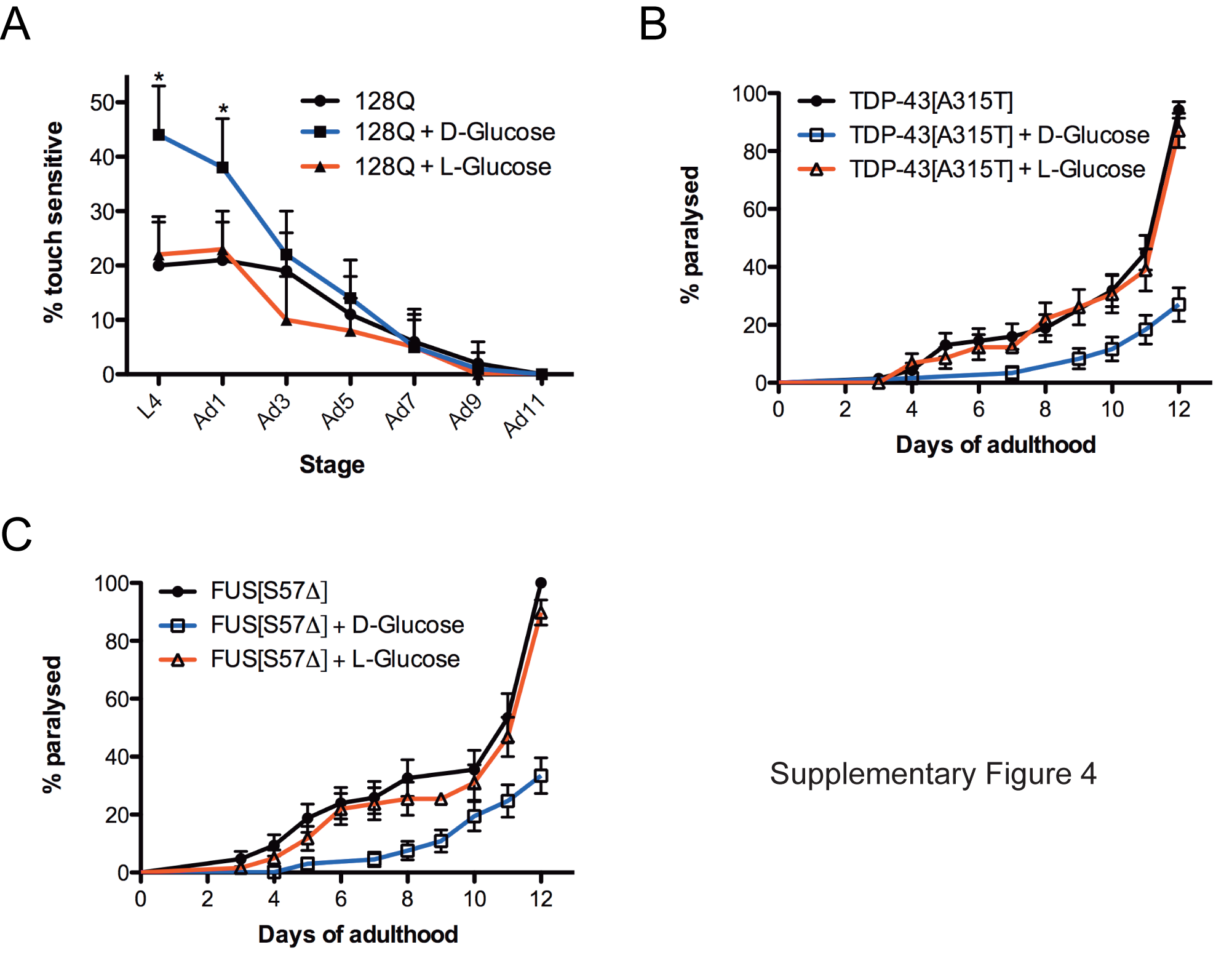


**Supplementary Figure 4.** L-glucose does not reduce neuronal proteotoxicity in *C. elegans* transgenics.

Animals were grown on 2% D-glucose, 2% L-glucose, or normal growth media from hatching and assayed for proteotoxicity through adulthood. (A) 2% D-glucose rescues touch insensitivity of 128Q animals at the larval L4 stage and day 1 of adulthood (*P < 0.01 versus untreated worms or animals treated with 2% L-glucose). (B) D-glucose rescues mTDP-43 induced paralysis compared to untreated worms or animals treated with 2% L-glucose (P<0.001). (C) D-glucose rescues mFUS induced paralysis compared to untreated worms or animals treated with 2% L-glucose (P<0.001)


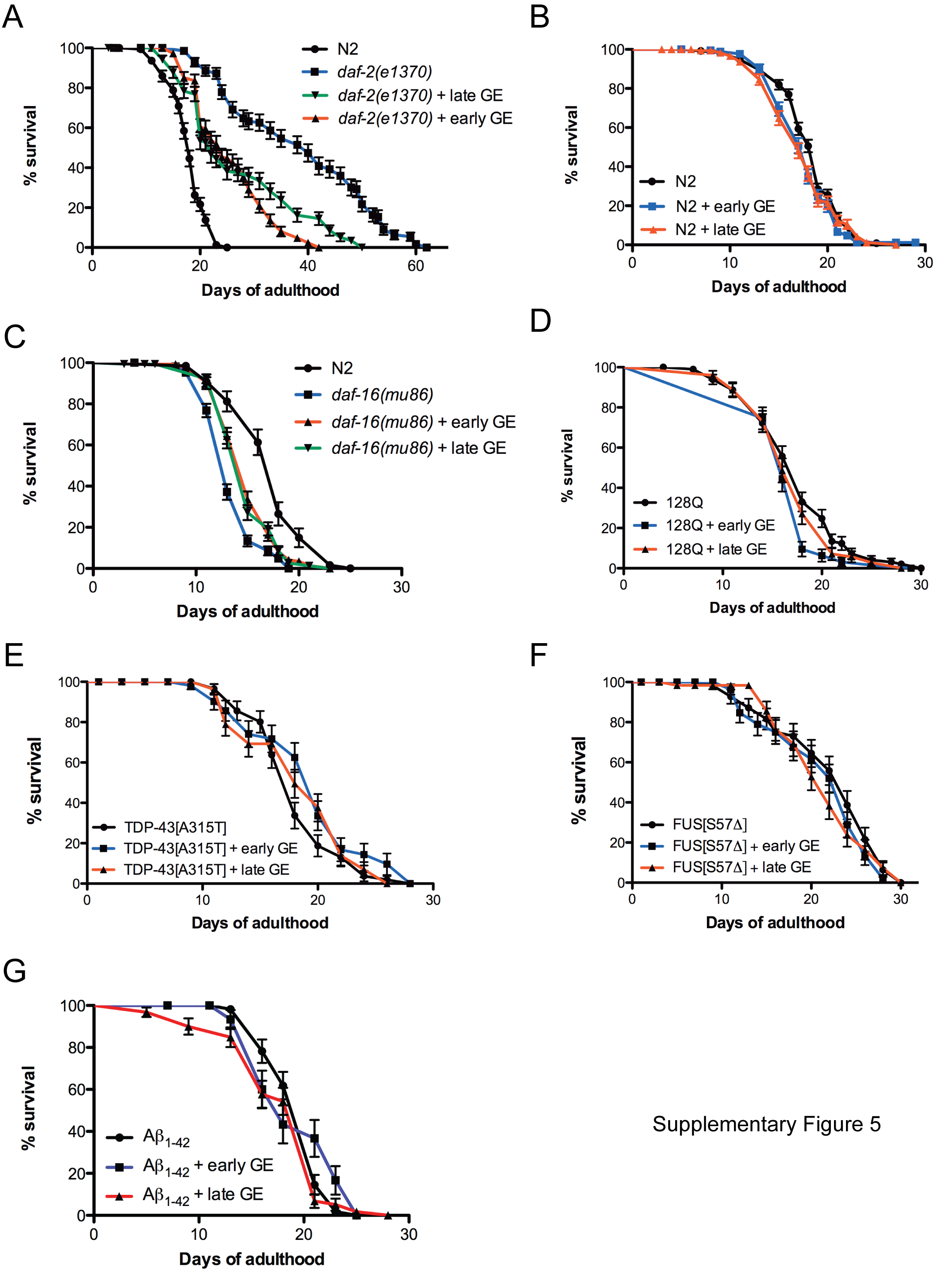


**Supplementary Figure 5.** GE reduces the lifespan of long-lived *daf-2* mutants but not strains with shorter lifespans. Lifespan was tested for multiple strains using either early or late GE (at 2% glucose). (A) Both early and late GE reduced the lifespan of long-lived *daf-2(e1370)* animals (P< 0.001 early or late GE versus untreated *daf-2* mutants). (B) The lifespan of wild type N2 animals was unaffected by early or late GE. (C) Neither early or late GE reduced the lifespan of *daf-16(mu86)* mutants. (D) There was no effect on lifespan by early or late GE for (D) 128Q, (E) mTDP-43, (F) mFUS or (G) β_1-42_ transgenics compared to untreated control strains.


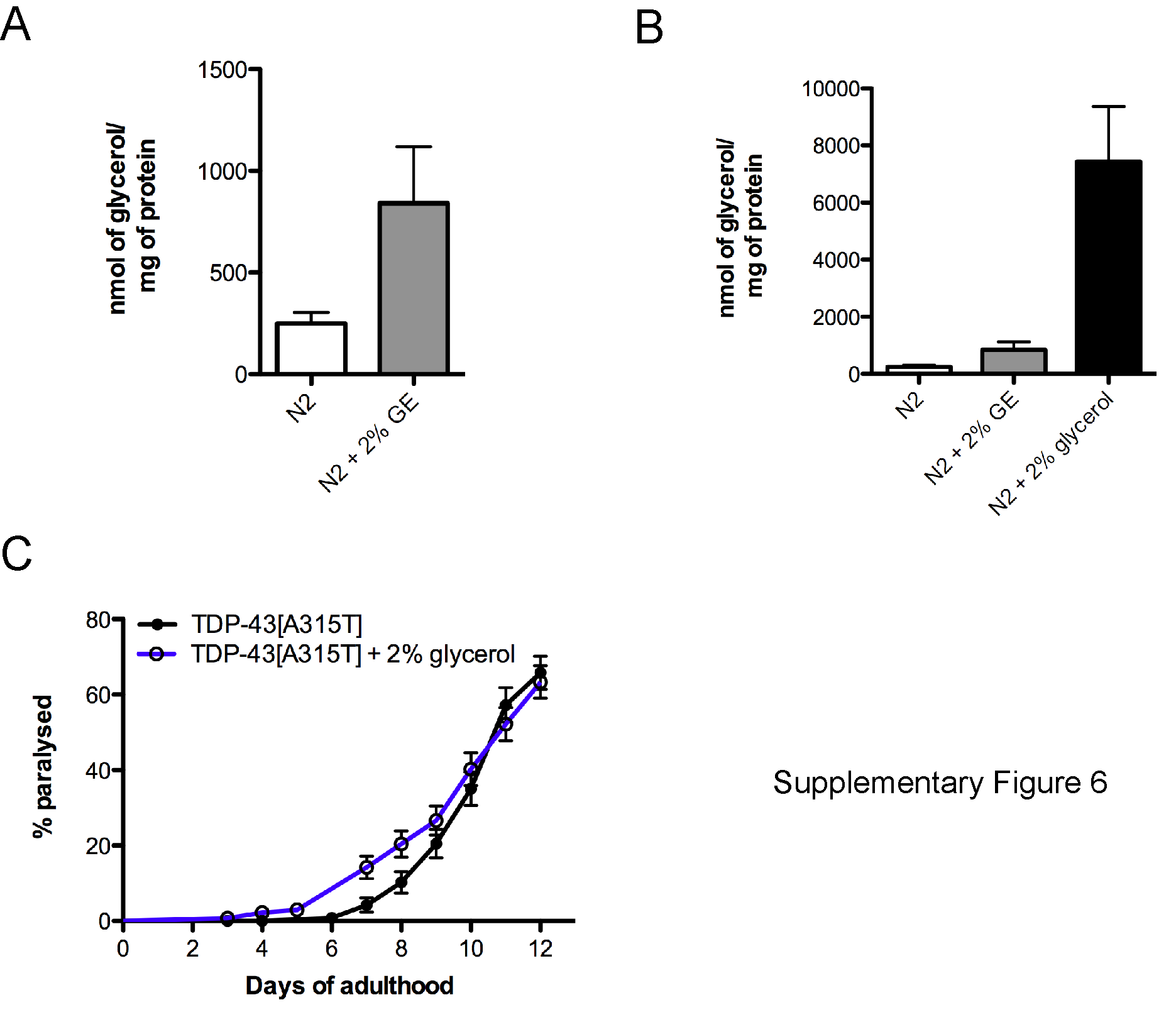


**Supplementary Figure 6.** Glycerol does not reduce mutant TDP-43 toxicity.

(A) N2 worms exposed to 2% glucose enrichment (GE) had increased internal glycerol levels as did (B) worms exposed to 2% glycerol. (C) 2% glycerol did not reduce mutant TDP-43 paralysis


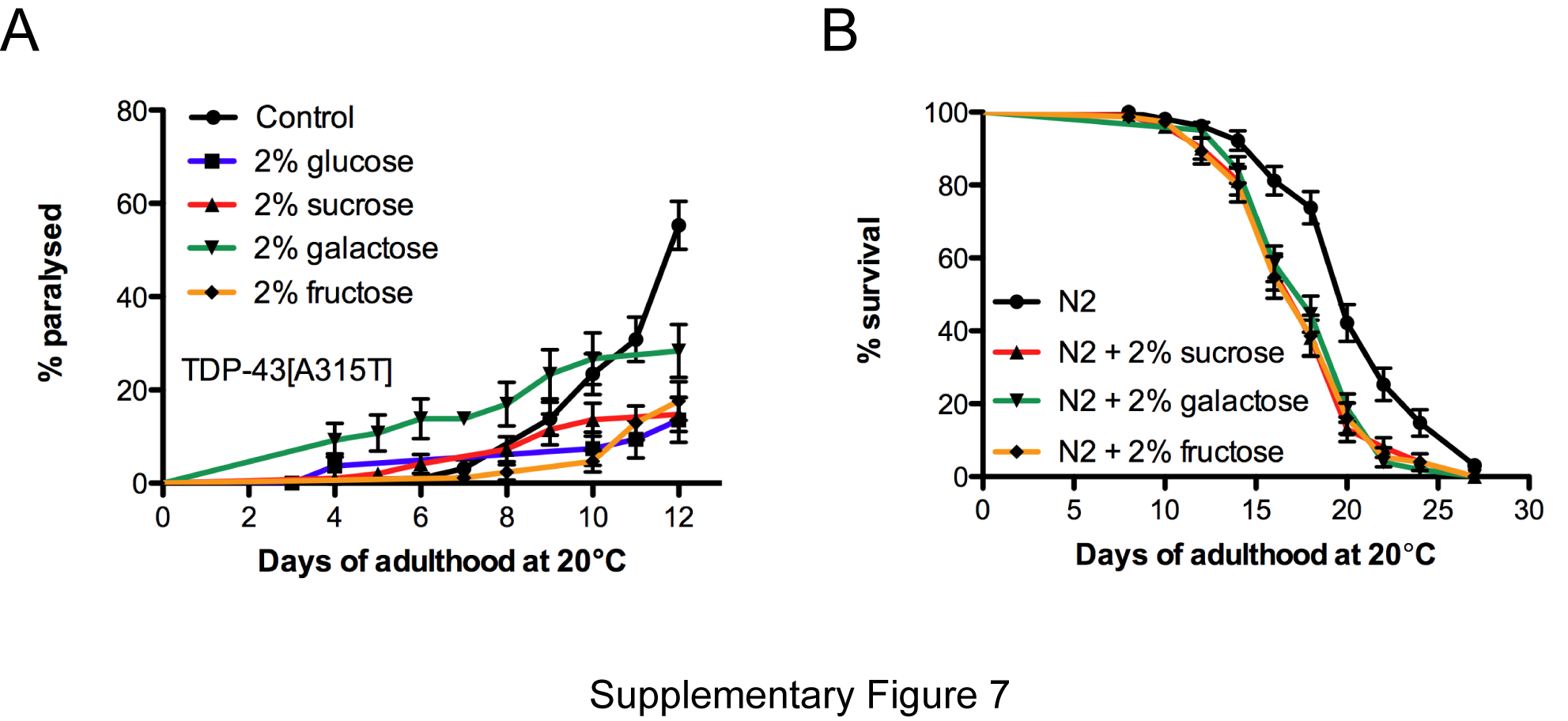


**Supplementary Figure 7.** Other sugars reduce proteotoxicity and lifespan.

(A) Mutant TDP-43 worms exposed to glucose, sucrose, galactose or fructose had significantly reduced rates of paralysis compared to untreated TDP-43 worms (P<0.001 for glucose, sucrose and fructose vs. untreated controls, P<0.01 for galactose vs. untreated controls). (B) All sugars tested reduced the lifespan of N2 worms compared to untreated controls.


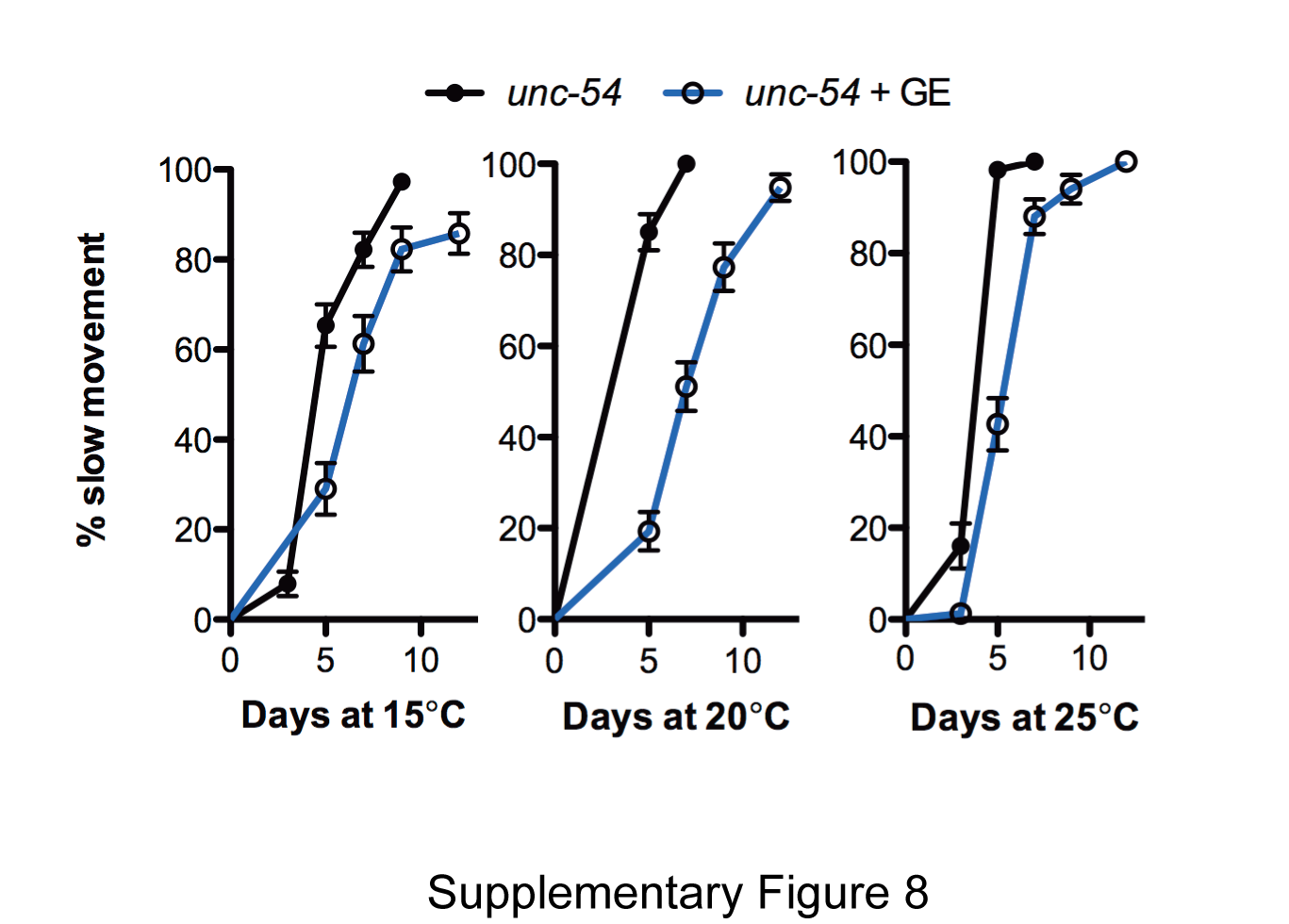


**Supplementary Figure 8.** Glucose rescues *unc-54* mutant phenotypes.

Glucose enrichment (GE) rescued the progressive slow movement phenotype of *unc-54* mutants at all temperatures tested (P<0.001 versus untreated *unc-54* mutants).


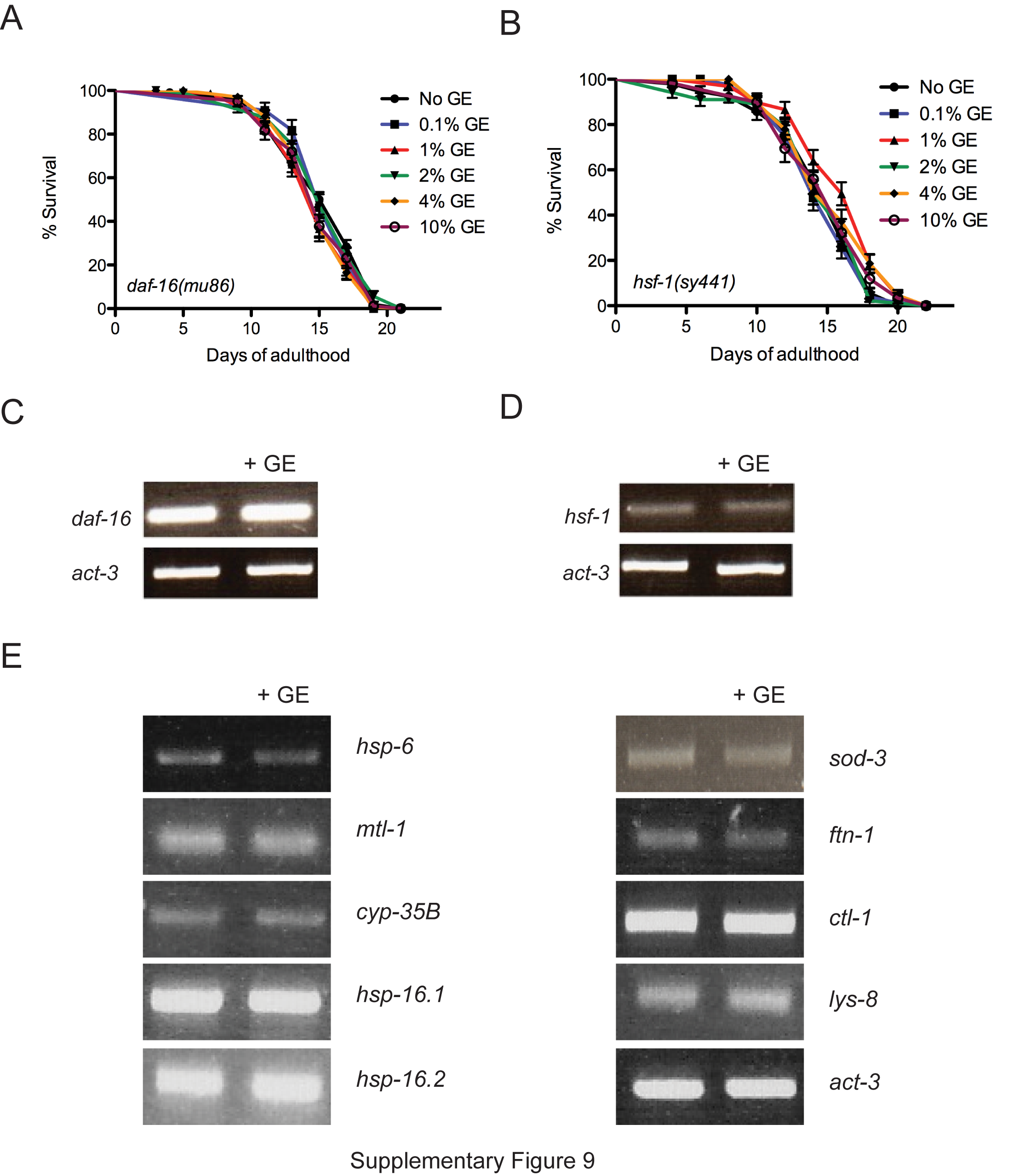


**Supplementary Figure 9.** Glucose does not affect *daf-16*, *hsf-1* or target gene expression.

Glucose enrichment (GE) at all concentrations did not reduce the lifespan of (A) *daf-16(mu86)* or (B) *hsf-1(sy441)* mutants. 2% GE did not alter the expression of (C) *daf-16* or (D) *hsf-1*. (E) 2% GE did not alter expression levels of a number of *daf-16* and *hsf-1* target genes. Hsp-6


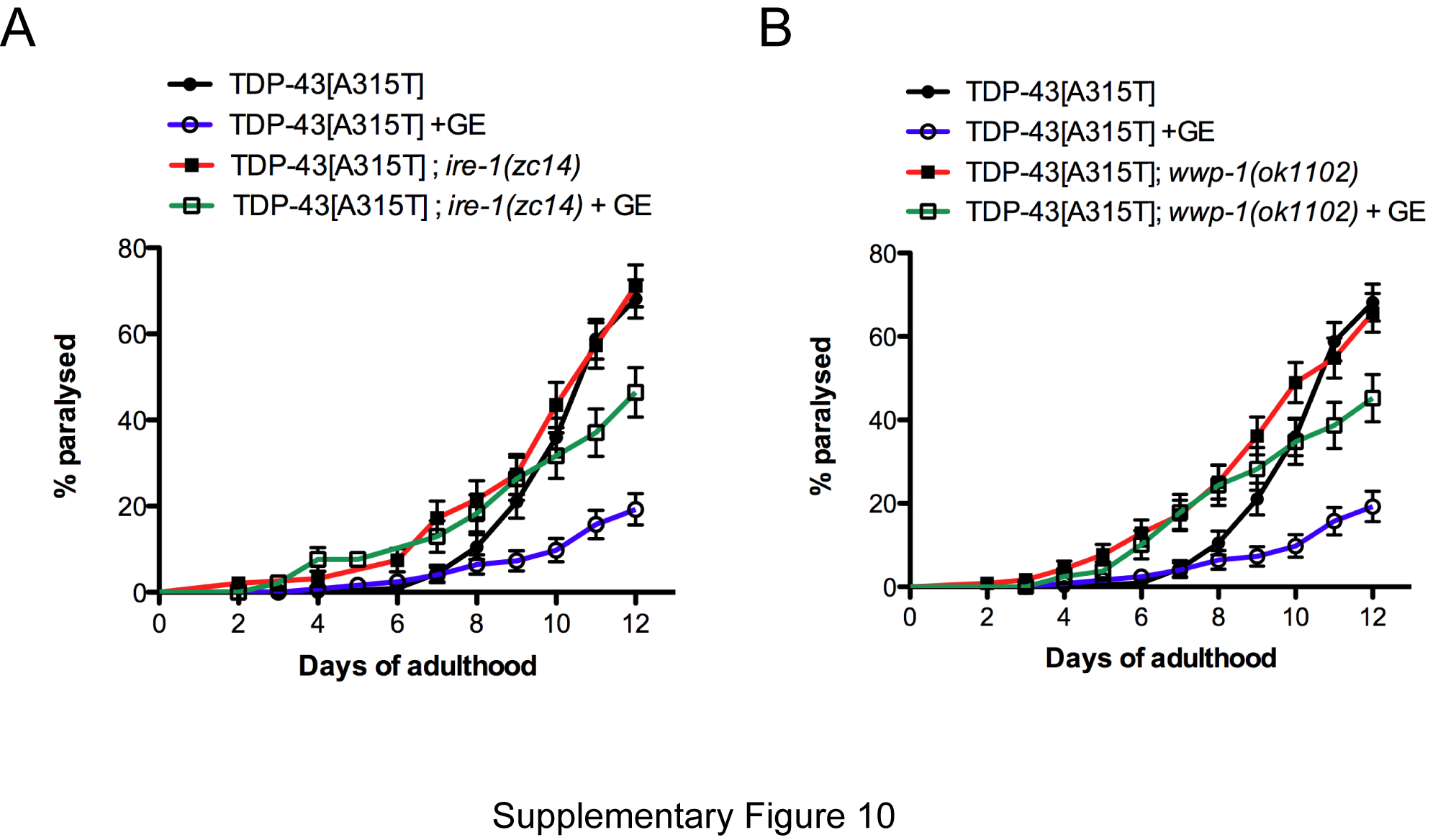


**Supplementary Figure 10.** Glucose neuroprotection requires protein homeostasis genes.

Glucose enrichment (GE) reduced mutant TDP-43 paralysis compared to untreated control strains but this was blocked by mutations in (A) *ire-1* or (B) *wwp-1.*

|  | **Strain** | **Mean Lifespan** | **p Value** | **75th Percentile (Days)** | **Maximum Lifespan** | **Total Number of Animals Died/Total** |
| --- | --- | --- | --- | --- | --- | --- |
| **Figure 1** | N2 | 19 |  | 21 | 26 | 49/65 |
|  | N2 + BD | 29 | <0.0001 | 36 | 40 | 34/80 |
| **Figure 4** | N2 | 17 |  | 20 | 25 | 138/142 |
|  | N2 + 0.1% | 17 | n.s. 0.9388 | 20 | 25 | 94/97 |
|  | N2 + 1% | 17 | n.s. 0.9333 | 20 | 25 | 87/96 |
|  | N2 + 2% | 17 | n.s 0.0741 | 20 | 25 | 99/101 |
|  | N2 + 4% | 15 | <0.0001 | 16 | 21 | 51/57 |
|  | N2 + 10% | 15 | <0.0001 | 16 | 21 | 59/65 |
|  | *daf-2(e1370)* | 40 |  | 45 | 54 | 73/112 |
|  | *daf-2(e1370)* + 0.1% | 30,5 | 0.0006 | 35 | 58 | 84/118 |
|  | *daf-2(e1370)* + 1% | 28 | <0.0001 | 32 | 41 | 79/108 |
|  | *daf-2(e1370)* + 2% | 27 | <0.0001 | 29.5 | 44 | 114/117 |
|  | *daf-2(e1370)* + 4% | 22 | <0.0001 | 24 | 43 | 72/85 |
|  | *daf-2(e1370)* + 10% | 18 | <0.0001 | 24 | 36 | 73/91 |
| **Figure S5** | *daf-2(e1370)* | 39 |  | 49.5 | 62 | 126/151 |
|  | *daf-2(e1370*) + EGE | 23.5 | <0.0001 | 30 | 41 | 138/155 |
|  | *daf-2(e1370)* + LGE | 21.5 | <0.0001 | 34 | 50 | 128/150 |
|  | *daf-16(mu86)* | 15 |  | 16 | 21 | 289/295 |
|  | *daf-16(mu86)* + EGE | 14 | n.s. 0,5832 | 16 | 23 | 147/149 |
|  | *daf-16(mu86)* + LGE | 14 | n.s. 0,2059 | 16 | 23 | 151/160 |
|  | N2 | 18 |  | 20 | 27 | 240/254 |
|  | N2 + EGE | 17 | n.s. 0.2639 | 18 | 29 | 165/176 |
|  | N2 + LGE | 17 | n.s 0.1415 | 17.5 | 27 | 166/189 |
|  | 128Q | 15 |  | 19 | 30 | 173/178 |
|  | 128Q + EGE | 16 | n.s. 0.0509 | 15 | 29 | 152/156 |
|  | 128Q + LGE | 16 | n.s. 0.2326 | 17 | 28 | 156/165 |
|  | TDP-43 [A315T] | 17 |  | 17 | 28 | 54/66 |
|  | TDP-43[A315T] + EGE | 19 | n.s. 0.0620 | 21 | 28 | 39/62 |
|  | TDP-43[A315T] +LGE | 18 | n.s. 0.3616 | 21 | 26 | 50/62 |
|  | FUS[S57Δ] | 13 |  | 26 | 30 | 48/63 |
|  | FUS[S57Δ] + EGE | 12 | n.s. 0.2750 | 24 | 28 | 51/63 |
|  | FUS[S57Δ] + LGE | 15 | n.s. 0.3745 | 24 | 30 | 54/62 |
|  | Aβ_1-42_ | 19,5 |  | 20 | 25 | 117/129 |
|  | Aβ_1-42_ + EGE | 17 | n.s. 0.5708 | 22 | 25 | 93/95 |
|  | Aβ_1-42_ + LGE | 19 | n.s. 0.2663 | 22 | 28 | 151/154 |
| **Figure S7** | N2 | 20 |  | 22 | 27 | 93/111 |
|  | N2 + 2% Sucrose | 18 | <0.0001 | 19 | 24 | 100/101 |
|  | N2 + 2% Galactose | 18 | <0.0001 | 19 | 24 | 101/101 |
|  | N2 + 2% Fructose | 18 | <0.0001 | 19 | 24 | 75/75 |
| **Figure S9** | *daf-16(mu86)* | 16 |  | 18 | 21 | 208/208 |
|  | *daf-16(mu86)* + 0.1% | 15 | n.s. 0.5812 | 16 | 19 | 65/66 |
|  | *daf-16(mu86)* + 1% | 15 | n.s. 0.4632 | 16 | 19 | 71/72 |
|  | *daf-16(mu86)* + 2% | 15 | n.s. 0.4587 | 16 | 21 | 89/90 |
|  | *daf-16(mu86)* + 4% | 15 | n.s. 0.3698 | 16 | 21 | 134/136 |
|  | *daf-16(mu86)*+ 10% | 15 | n.s. 0.3333 | 16 | 21 | 82/82 |
|  | *hsf-1(sy441)* | 16 |  | 17 | 20 | 90/98 |
|  | *hsf-1(sy441)* + 0.1% | 14 | n.s. 0.0745 | 16 | 22 | 78/89 |
|  | *hsf-1(sy441)* + 1% | 16 | n.s. 0.0901 | 17 | 22 | 97/101 |
|  | *hsf-1(sy441)* + 2% | 14 | n.s. 0.4955 | 15 | 22 | 91/102 |
|  | *hsf-1(sy441)* + 4% | 15 | n.s. 0.4021 | 17 | 22 | 86/98 |
|  | *hsf-1(sy441)* + 10% | 16 | n.s. 0.3952 | 17 | 22 | 59/59 |

**Supplementary Table 1.** Lifespan analysis for all experiments. Related to Figure 4, and Supplementary Figures , 7 and 8 and 8.

Animals that died prematurely (ruptured, internal hatching) or were lost (crawled off the plate) were censored at the time of scoring. All control and experimental animals were scored and transferred to new plates at the same time. n.s. not significant.
